# Emerging Materials and Technologies with Applications in Flexible Neural Implants: A Comprehensive Review of Current Issues with Neural Devices

**DOI:** 10.1002/adma.202005786

**Published:** 2021-05-29

**Authors:** Younguk Cho, Sanghoon Park, Juyoung Lee, Ki Jun Yu

**Affiliations:** ^1^ School of Electrical Engineering Yonsei University Seoul 03722 Korea; ^2^ School of Electrical Engineering YU‐KIST Institute Yonsei University Seoul 03722 Korea

**Keywords:** biointegrated electronics, flexible electronics, neural implants

## Abstract

Neuroscience is an essential field of investigation that reveals the identity of human beings, with a comprehensive understanding of advanced mental activities, through the study of neurobiological structures and functions. Fully understanding the neurotransmission system that allows for connectivity among neuronal circuits has paved the way for the development of treatments for neurodegenerative diseases such as Parkinson's disease, Alzheimer's disease, and depression. The field of flexible implants has attracted increasing interest mainly to overcome the mechanical mismatch between rigid electrode materials and soft neural tissues, enabling precise measurements of neural signals from conformal contact. Here, the current issues of flexible neural implants (chronic device failure, non‐bioresorbable electronics, low‐density electrode arrays, among others are summarized) by presenting material candidates and designs to address each challenge. Furthermore, the latest investigations associated with the aforementioned issues are also introduced, including suggestions for ideal neural implants. In terms of the future direction of these advances, designing flexible devices would provide new opportunities for the study of brain–machine interfaces or brain–computer interfaces as part of locomotion through brain signals, and for the treatment of neurodegenerative diseases.

## Graphical abstract

Flexible implants have attracted increasing interest, mainly to overcome the mechanical mismatch between rigid materials and soft neural tissues, enabling precise measurements of neural signals from conformal contact. The current issues of flexible neural implants are summarized by presenting material candidates and designs to address challenges. Furthermore, the latest investigations associated with each issue are also introduced.

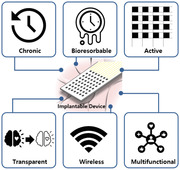

## 1 Introduction

Recent implantable neural devices have received significant attention because they can provide unprecedented cures for patients suffering from neurodegenerative disease or chronic pain.^[^ ^1^ ^]^ These devices are not only applied to achieve certain clinical objectives, but also serve to generate big data regarding the stream of neural signals through a brain–computer interface (BCI) or brain–machine interface (BMI).^[^ ^2^ ^]^ Among them, implantable devices with high flexibility offer distinct benefits and accurate monitoring of physiological signals by establishing conformal contact between the devices and tissues and accommodating the applied strain from the deformable geometry of the target organs, thereby alleviating device failures.^[^ ^3^ ^]^ A fundamental consideration for desirable implants is to acquire or modulate neurodynamic signals from the body as much as possible without the background noise. The external noise includes the photoelectric effect during light stimulation,^[^ ^4^ ^]^ motion‐induced signal artefacts,^[^ ^5^ ^]^ and electrode insulation caused by an immune response.^[^ ^6^ ^]^ To date, multiple strategies have been employed to address the aforementioned issues.^[^ ^7^ ^]^ Nevertheless, there are still obstacles that limit the successful recording/modulating platforms in terms of the material properties and technical methods of device fabrication.

This article discusses the current issues and challenges of recent neural implants and suggests future research directions. First, an overview of the nervous system with clinically implantable organs is presented in Section 2. This section also reviews the latest research on the functions of the device inserted in each organ. The review highlights the problems in subsequent sections in six parts: 1) nonchronic electrodes, 2) non‐bioresorbable electronics, 3) passive electrode arrays, 4) non‐transparent electrode arrays, 5) externally wired electronics, and 6) monofunctional devices. Several approaches to address these problems are presented in Section 4 with the associated studies. The Conclusion section (Section 5) highlights the future neuro‐implantable devices that leverage neuroscience research with clinical applications.

## 2 Overview of the Nervous System with Recent Neural Implants

**Figure 1:**
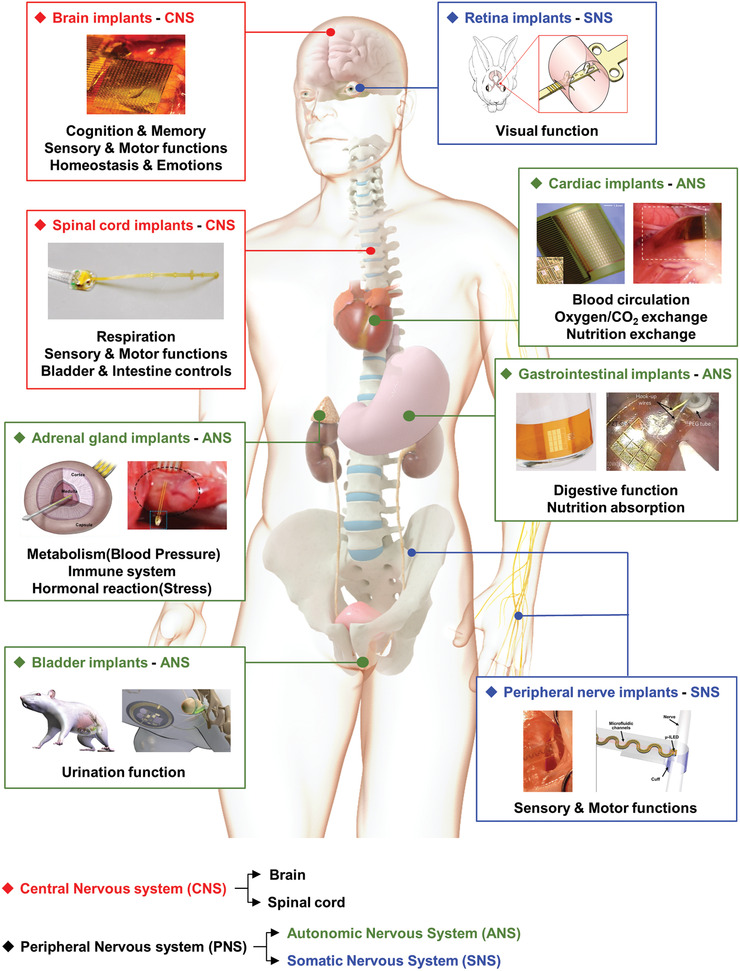
Schematic illustration of neural implants applied to nervous systems. Image for “Brain implants”: Reproduced with permission.^[^ ^13b^ ^]^ Copyright 2011, Springer Nature. Image for “Spinal cord implants”: Reproduced with permission.^[^ ^17^ ^]^ Copyright 2016, Springer Nature. Image for “Retina implants”: Reproduced with permission.^[^ ^19^ ^]^ Copyright 2019, The Authors, published by Springer Nature. Image for “Peripheral nerve implants”: Reproduced with permission.^[^ ^21^ ^]^ Copyright 2019, The Authors, published by American Association for the Advancement of Science (AAAS). Reprinted/adapted from ref. ^[^ ^21^ ^]^. © The Authors, some rights reserved; exclusive licensee American Association for the Advancement of Science. Distributed under a Creative Commons Attribution NonCommercial License 4.0 (CC BY‐NC) http://creativecommons.org/licenses/by-nc/4.0/. Image for “Cardiac implants”: Reproduced with permission.^[^ ^23^ ^]^ Copyright 2010, American Association for the Advancement of Science. Image for “Adrenal gland implants”: Reproduced with permission.^[^ ^24^ ^]^ Copyright 2019, The Authors, published by National Academy of Sciences, USA. Image for “Gastrointestinal implants”: Reproduced with permission.^[^ ^25c^ ^]^ Copyright 2017, The Authors, published by Springer Nature. Image for “Bladder implants”: Reproduced with permission.^[^ ^25b^ ^]^ Copyright 2019, Springer Nature.

The nervous system plays an important role as a control network through processing biological information or transmitting sensory expressions.^[^ ^8^ ^]^ The nervous system is divided into the central nervous system (CNS) and the peripheral nervous system (PNS), and each has subdivisions. The detailed schematic illustrations are shown in **Figure** **1**.

The CNS comprises the brain and spinal cord, where sensory information from the PNS following behavioral responses is collected and processed.^[^ ^9^ ^]^ The brain engages in cognition, memory,^[^ ^10^ ^]^ and language skills as well as maintaining homeostasis through hormonal control.^[^ ^11^ ^]^ The external part of the cerebral cortex consists of gray matter with a collection of nerve cells. The dynamics of the brain are synchronized with cardiac cycles and respiration, thereby transmitting information throughout the body.

Different types of brain implants have recently been developed to identify encoded data in the action potential and thereby elucidate how the neuronal circuit functions.^[^ ^12^ ^]^ These devices accommodate electrical signals from neural tissues by storing them on a subdural space, penetrating target cells, or injecting them deep in the brain.^[^ ^13^ ^]^ Some studies have simultaneously performed intracranial pressure investigation and brain temperature measurement, demonstrating the close relationship between neural activity and the physical properties of the brain.^[^ ^14^ ^]^

The spinal cord receives information about changes in and out of the body from the peripheral nerves and transmits it to the brain. In particular, the neuronal traces that travel down from the brain are responsible for respiration control and locomotion.^[^ ^15^ ^]^ Therefore, recording electrophysiological signals from the spinal cord provides options for the treatment of patients who suffer from gait disturbance or traumatic injury.^[^ ^16^ ^]^ As a result, this recording process has a significant impact on the design of a therapeutic device for spinal cord injuries.^[^ ^17^ ^]^

The PNS is connected to nerves from the CNS and is spread throughout the body, conveying information from the sensory muscles and involuntary functions.^[^ ^18^ ^]^ In the case of retinal implants, research has mainly focused on elucidating the relationship between the visual cortex and retina by stimulating the optic nerves.^[^ ^19^ ^]^ The somatic nervous system and autonomic nervous system are representative subdivisions of the PNS, depending on the direction of signal propagation. Because the somatic efferent nerves include neurons that transmit excitation from the CNS, most implantable devices are designed for the replacement of a sensory function or nerve regeneration.^[^ ^20^ ^]^ Flexible cuff electronics are also introduced to interrogate electrical signals during motor function.^[^ ^21^ ^]^ The autonomic nervous system innervates the organs and intestines, allowing them to voluntarily control physical conditions such as respiration and heart function.^[^ ^22^ ^]^ Recently developed flexible cardiac implants help scrutinize action potentials from the sinoatrial node in real time.^[^ ^23^ ^]^ Furthermore, devices for treating the abnormal rhythm of the heart, such as the conventional pacemaker, have been designed. Another example is a probe inserted into the adrenal medulla, which enables stress monitoring by analyzing electrophysiological signals, relying on the concentration of adrenocorticotropic hormone.^[^ ^24^ ^]^ Devices can also be implanted into the bladder or gastrointestinal tract such as actuators and nanogenerators, utilizing the properties of voluntary contraction and relaxation cycles.^[^ ^25^ ^]^

Neuro‐prosthetics for recording or modulating physiological signals from different parts of the body have, in essence, been designed for functional exploitation. However, there are still several challenges during data acquisition regardless of the insertion site. In the following sections, several issues are identified in conventional implantable devices and the advantages of solving these problems are discussed.

## 3 Problems and Challenges of Flexible Neural Implants

### 3.1 Nonchronic and Non‐Bioresorbable Electronics

One rudimentary consideration for neuro‐prosthetics is whether they provide biological signals in the long term, bypassing immune responses from the body.^[^ ^26^ ^]^ However, insertion of the implantable device itself causes several problems for the host body. First, a penetrating probe passing through tissue surfaces gives rise to tissue wound by damaging blood vessels. Direct contact of the implantation site with a device, such as an electrocorticogram (ECoG), results in a mechanical mismatch between the tissue and the device applying constant pressure to the nerve cells. The immune response caused by an implantable device is called a “foreign body reaction” (FBR).^[^ ^27^ ^]^ As the device is inserted into the tissue, protein adhesion to the device, called “acute inflammatory response,” is immediately accelerated. Protein adhesion due to the process of phagocytosis specifically occurs in the wound area by generating blood scabs. Then, it forms a provisional matrix on the surface of the tissue. Subsequently, the immune cells adhere to the wound site and continuously influence the surrounding tissues to increase the range of infection. Eventually, a foreign body giant cell and fibrous encapsulation formation appear in the area around the wound.

**Figure 2:**
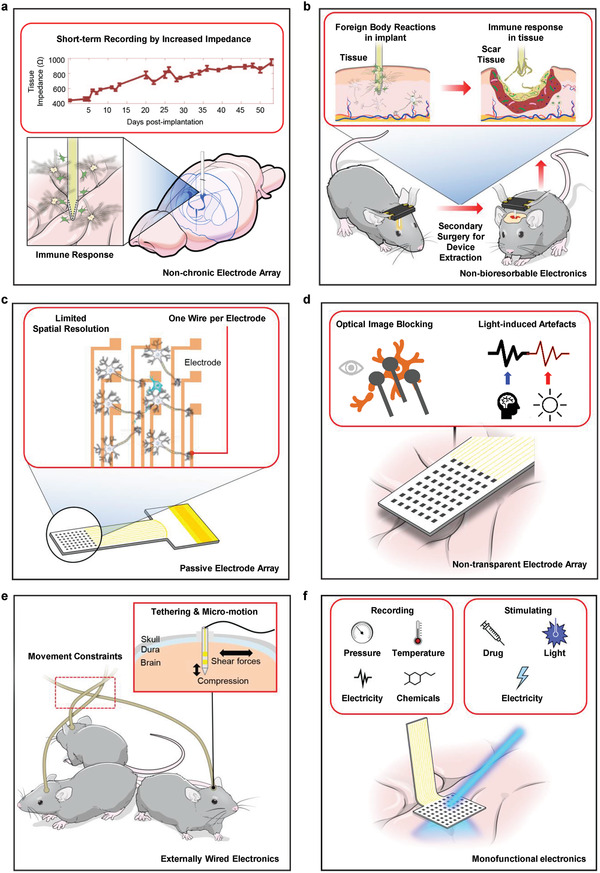
Recent issues and challenges of neural implants. a) Possible challenges of nonchronic electrode arrays (limitation with short‐term recording by increased impedance and immune response). b) Possible challenges of non‐bioresorbable electronics (secondary surgery for device extraction and foreign body reaction in implant). c) Possible challenges of passive electrode array (limitation of spatial resolution and one wire per electrode). d) Possible challenges of non‐transparent electrode array (optical image blocking and light‐induced artefacts). e) Possible challenges of externally wired electronics (movement constraints and tethering and micromotion). f) Possible challenges of monofunctional electronics (complex and inaccurate measurement). a) Reproduced with permission.^[^ ^28b^ ^]^ Copyright 2016, IPEM, published by Elsevier Ltd.

A nonchronic device that does not prevent cell adhesion after implantation encounters an inflammatory reaction on the surface of the implanted device, which leads to device failure by continuous growth of fibrous cells. The formation of fibrous capsules on the electrode array increases the impedance of the sensing pad, thereby deteriorating the device's sensitivity and reliability, as shown in **Figure** **2**a.^[^ ^28^ ^]^ An implanted electrode can no longer record or modulate electrophysiological signals after being fully covered with fibrous cells, and additional surgery is needed to replace the device.^[^ ^29^ ^]^

However, performing a secondary surgery to replace or remove the implanted device causes further infections. Previously formed fibrous cells cover the overall exterior parts of the device, generating wound tissue. As a result of this fully covered immune responses, additional surgery to remove the implanted device causes the extraction of near tissues, as shown in Figure 2b. This incident leads to additional inflammatory response and infections. Moreover, clinical devices such as pacemakers or cochlear implants should guarantee permanent operation for patients without device failure. Nonetheless conventional implant devices are not bioresorbable devices that become useless after the desired operation time.^[^ ^30^ ^]^ Continuous contact of the external components with the body prompts infection in the organs. Therefore, a secondary surgery to remove the device becomes necessary for the patient's healthcare. Such extra surgery significantly increases the risk to the patient, leading to additional physical and financial burden.^[^ ^31^ ^]^

### 3.2 Passive Electrode Arrays

Passive electrode arrays have limitations for a large area neural recording with high‐density. High resolution and a large‐area neural interface by recording many single‐unit action potentials are important to understand the complex interactions in neurons.^[^ ^32^ ^]^ However, the property that a passive device directly measures the electrical signal generated by the tissue without active components such as amplifiers or multiplexed transistors limits a device in which one wire must be connected per channel. As a result, manufacturing many high‐density electrodes is difficult due to the increased number of wires corresponding to the increased number of electrodes. Furthermore, low temporal resolution remains a challenge because of the interconnected lines passing through the electrodes at the end of the device in external equipment. As shown in Figure 2c, unrecorded neurons can exist under interconnect lines, reducing the accuracy of the neuron signal pathway. As a result of these limitations, passive neural electronics cannot be developed for a high‐density, high spatial resolution device, which is significantly important in neural electrophysiological recording.

### 3.3 Non‐Transparent Electrode Arrays

An electrophysiological measurement system has the compelling advantage of possible direct interpretation without a subsequent translation process. However, direct contact between a recording electrode and a biological tissue causes interference in neuronal dynamics.^[^ ^33^ ^]^ Limited spatial resolution because of the restricted number of electrodes and recording sites from the implants is an added drawback of electrical sensing modality. Methodological integration of an electrophysiological recording and imaging technique has led to a fundamental shift in biomedical engineering and the field of neuroscience.^[^ ^34^ ^]^ For instance, magnetic resonance imaging (MRI) compatible devices exhibit the strengths of both the electrophysiological signal measurement method and brain imaging.^[^ ^35^ ^]^ Optical approaches offer critical benefits such as outstanding spatial resolution, elimination of direct contact with cells, and real‐time investigation of tissue behavior. Better spatiotemporal resolution of neural recording is obtained in combination with an electrical signal readout in terms of the ability to identify the relationship between electrophysiological signals and tissue morphologies.^[^ ^36^ ^]^ Ultimately, this functional incorporation provides experimental data to determine the causality between the “structures” and “functions” of neurons. An opaque electrode array for recording biological signals from neural cells impedes the synergism of combining electrical and optical modalities. The non‐transparent readout site blocks most of the field of view from the underlying population of neurons, hampering querying of the optical microscopy database.^[^ ^37^ ^]^

Light‐induced artefacts created by light‐impenetrable electrodes pose severe challenges to interpreting nerve cells signals.^[^ ^4^ ^]^ These artefacts are interpreted as a photoelectric effect, indicating unintended voltage or current generated from the electrode array upon exposure to light. Photoactivation in neural implants occurs in the form of mechanistically different modes, such as the photovoltaic mode, photoconductive mode, and photothermal mode. Among these, the photovoltaic mode is the most influential mechanism during the electrophysiological readout process. The photovoltaic mode indicates that an electron's energy level is excited by photons generating voltage or charge without electrical field bias. In particular, an electrical artefact in the form of voltage is similar to a single‐unit potential from a neuron. Corrupted data generated from artefacts clearly highlight the need to use an alternative instead of opaque metal‐based electrode arrays, as shown in Figure 2d.

### 3.4 Externally Wired Electronics

Conventional neural recordings or modulation systems include a device, external connections with data acquisition hardware, and monitoring devices. In particular, in a neuro‐modulation system, conductive wires and optical fibers are an essential configuration to transmit external stimuli directly to the nerve cells.^[^ ^38^ ^]^ In the case of a fully implantable device, the device itself is entirely inside the body, whereas the lines connected to the external machine remain on the exterior. Behavioral studies from animal models under natural conditions are indispensable in the field of neuroscience.^[^ ^39^ ^]^ However, externally wired implants frustrate freely moving animals, causing continuous irritation and constraints.^[^ ^40^ ^]^

Physical implants tethered with a wire into the brain also exhibit implant failure, as shown in Figure 2e.^[^ ^41^ ^]^ The behaviors of experimental animals such as head rotation, wandering around the cage, or nose poking produce shear force and compression of devices fixed at the intracranial space.^[^ ^42^ ^]^ Micromotion due to these forces eventually results in functional damage of the brain.^[^ ^43^ ^]^ In its entirely enclosed state within the skull, the brain is separated from external mechanical forces because it remains floating in the cerebrospinal fluid (CSF). In the device‐implanted state, however, brain displacements apply repeated stress on the interface due to the partially enclosed area of the brain as a result of the previously mentioned forces. In the case of flexible tethers, the shear forces and torques are lower than those of a rigid one. Nevertheless, they are not completely free from problems because of externally protruding components including wires. Thus, there are strong demands for completely implantable wireless devices that eliminate chronic abrasion from tethered operation using miniaturized LEDs when combining optogenetic approaches.

### 3.5 Monofunctional Electronics

A sensing platform for monitoring patients’ conditions and strategies for therapeutic neuromodulation are needed to rehabilitate patients who suffer from neurological disorders.^[^ ^44^ ^]^ For an ideal treatment with respect to pathology, it is necessary to find the exact lesions contributing to a neurodegenerative disease with pinpoint stimuli in the same area. However, the conventional monofunctional device simply has one function of neural sensing or stimulation; therefore, extra supplementary equipment for integrated drug delivery, optoelectronics, and an electrode array for electrical activation are necessary, as shown in Figure 2f. Furthermore, the supporting equipment creates a complicated experimental environment due to the external wire connections, thereby contaminating biological data with user discomfort. The separation of sensing and stimulating devices also requires a calibration process that adjusts the time difference between them. Thus, it is impossible to create a closed loop (feedback system) that applies to the stimulation at the exact time. The decrease in spatial resolution also originated from the positional difference between the sensing site and the modulating point. Accordingly, neuronal activity inhibition or excitement occurs in an unwanted area, and not where the electrophysiological signals are acquired. Consequently, this temporal and spatial mismatch caused by the monofunctional device degrades the quality and reliability of the experimental results.^[^ ^45^ ^]^

## 4 Addressing the Issues and Challenges Posed by Flexible Neural Implants

### 4.1 Chronic Stable Electrode Arrays

It is important to measure electrophysiological signals and stimulate the brain in the long term to determine more accurate correlations and propagation between neuronal signals related to neurological diseases.^[^ ^32b^ ^]^ It is recommended that the size of scars occurring at the insertion sites of bioelectronics into the tissue should be reduced prior to recording stable neuronal dynamics over a long period of time.^[^ ^26^ ^]^ This approach ensures that neurons do not recognize the device as an external substance so as not to induce any immune responses. There are two ways to meet the above requirements for long‐term recording, namely using structurally biocompatible devices and encapsulation of biocompatible materials in the devices.

#### 4.1.1 Structural Biocompatibility for Long‐Term Recording

**Figure 3:**
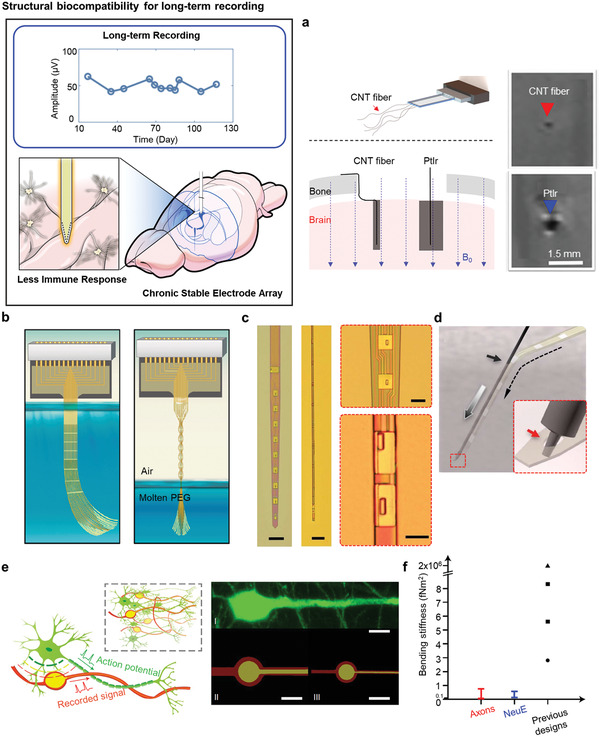
Expected advantages of chronic electrode array. Structural biocompatibility for long‐term recording. a) Schematic illustration of soft and MRI compatible neural electrodes from carbon nanotube fibers (top). Optical image of MRI artifacts of implanted CNT fiber and PtIr electrodes on horizontal sections of a rat brain (bottom). Reproduced with permission.^[^ ^46^ ^]^ Copyright 2019, American Chemical Society. b) Schematics of elastocapillary self‐assembled neurotassels. Reproduced with permission.^[^ ^47^ ^]^ Copyright 2019, The Authors, published by American Association for the Advancement of Science (AAAS). Reprinted/adapted from ref. ^[^ ^47^ ^]^. © The Authors, some rights reserved; exclusive licensee American Association for the Advancement of Science. Distributed under a Creative Commons Attribution NonCommercial License 4.0 (CC BY‐NC) http://creativecommons.org/licenses/by-nc/4.0/. c) Optical image of NET‐50 (left, scale bar: 100 µm), and NET‐10 (middle, scale bar: 50 µm) with a magnified view of NET‐50 (right‐top) and NET‐10 (right‐bottom). Scale bar: 10 µm. d) Schematic diagram of insertion mechanism of ultrathin neural probe using a shuttle device. Inset shows that the shuttle is used in the microhole on the probe. c,d) Reproduced with permission.^[^ ^49^ ^]^ Copyright 2017, The Authors, published by American Association for the Advancement of Science (AAAS). Reprinted/adapted from ref. [49]. © The Authors, some rights reserved; exclusive licensee American Association for the Advancement of Science. Distributed under a Creative Commons Attribution NonCommercial License 4.0 (CC BY‐NC) http://creativecommons.org/licenses/by-nc/4.0/. e) Schematic diagram of neuron‐like electronics (NeuE); the neurons show structural similarity. Inset shows a neuron network with NeuE at the subcellular level (left). Fluorescence microscopy images of a neuron (I, right) and scanning electron microscopy (SEM) images of NeuE designs (II and III). Scale bar: 10 µm. f) Bending stiffness of axons, NeuE, and previous designs that include state‐of‐the‐art mesh (circle), fiber (triangle), and thread probes (squares). e,f) Reproduced with permission.^[^ ^7d^ ^]^ Copyright 2019, The Authors, published by Springer Nature.

Low bending stiffness and ultraflexibility, similar to the mechanical, structural properties of neurons, lead to chronic recording for neural implants. **Figure** **3**a shows a carbon nanotube (CNT) fiber electrode used to measure electrophysiological signals for 6–12 weeks with significantly smaller amplitude of artefacts compared with using a PtIr electrode.^[^ ^46^ ^]^ A soft CNT fiber with a diameter of 10 µm was inserted with a tungsten wire shuttle with a diameter of 50 µm attached using a polyethylene oxide (PEO) adhesive. The use of this shuttle insertion method significantly improved the footprint of the brain in terms of the size compared with that of the existing PtIr electrode. With reduced diameter and bending stiffness, the density of the astrocytes and microglia around the inserted electrode position was significantly decreased compared with the PtIr electrode, and the deteriorated area of neurons also decreased over time, allowing for long‐term recording. Furthermore, it was confirmed that the amplitude of artefacts during MRI measurement also drastically decreased compared with those of the existing electrodes, offering the capability of simultaneous long‐term recording with the electrode still inserted.

Besides, the main problem with the flexible neural probe for chronic recording is that it is difficult to insert because of its low bending stiffness. To this end, a method of measuring the electrophysiological signal using assembled thin filaments solidified with polyethylene glycol (PEG) has been studied.^[^ ^47^ ^]^ A 16‐channel electrode with a total thickness of 0.1–3 µm and a bending stiffness of <0.1 nN m was manufactured, and the probes were collected with elastocapillary self‐assembly using molten PEG to create a diameter of 55 µm to allow insertion into the brain. Figure 3b shows the neuro‐tassel immersed in molten PEG and then slowly lifted up to achieve a certain stiffness after drying in air. When inserted into the brain in this state, the solidified PEG slowly melts, making it possible to measure the action potentials and local field potentials (LFPs) that indicate the learning‐related activation in the medial prefrontal cortex (mPFC). This device was also designed to be used for measurement for 4–6 weeks with a low modulus, as well as a small insertion point and a diameter of 50 µm. Unlike conventional silicon probes,^[^ ^42^, ^48^ ^]^ the neuronal cell loss occurring around the probe was significantly reduced over time.

Furthermore, there is another case in which the bending stiffness is reduced to the nano‐Newton range by making the entire thickness and width of the probe thin, thereby making it similar to a single‐cell traction force. The ultraflexible nanoelectronic thread (NET) brain probes shown in Figure 3c,d were manufactured with a thickness of 1 µm and an average width of 50 µm for the NET‐50, and a thickness of 1.5 µm and a width of 10 µm for the NET‐10.^[^ ^49^ ^]^ The probes can be inserted through the microhole of each device to the desired depth using carbon fibers. As a result, the overall insertion footprint was ≈10 µm across, which resulted in a cell‐sized surgical damage with minimal bleeding and a small insertion force. It was confirmed that the immune response did not occur by normal density and morphology of microglial around the probe and the presence of astrocytes showed normal activity after implantation for 5 months. When scanning the blood flow rate, the rate near the probe and the rate far from the probe were similar, which indicates that the vascular and cellular structures of the tissue were maintained after implantation.

If an implanted device has similar structural and mechanical properties as neurons such that the device is not recognized as a foreign material, the immune responses can be significantly reduced. The bioinspired design of the neural probe with mesh structures was inserted using the syringe‐injection method to perform continuous electrophysiological measurements for 4 months.^[^ ^7d^ ^]^ It was confirmed that the microglial or astrocytes remained in their normal state. Probes fabricated to have structurally similar properties of neurons contain the electrode configuration and interconnects in the form of a soma and neurite. The insulation layer covered with a thin polymer, similar to the myelin sheath of a neuron, is also shown in Figure 3e. The mechanical stiffness also decreased by a factor of 20 compared with that of the existing neural probes, yielding a device thickness of 0.9 µm and width of 5 µm, similar to those of an axon, as shown in Figure 3f. The polymer encapsulation layer protects the device layer such that the device can continuously measure neural signals for 4 months, maintaining low impedance variations. In the case of continuous recording of a single neuron signal, additional neuron signals were captured at the same channel, which indicates that newborn neurons were clearly formed. It was confirmed through 3D image mapping using the mesh electrodes as a substrate that newborn neurons were formed.

#### 4.1.2 Representative Materials for Long‐Term Recording

**Figure 4:**
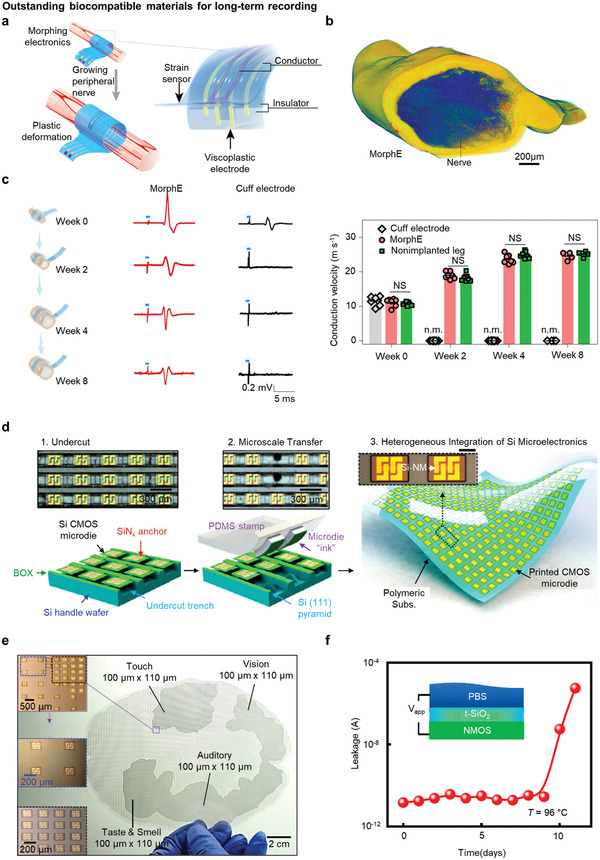
Outstanding biocompatible materials for long‐term recording. a) Schematic illustration of the mechanism of the morphing electronics that can be deformed when wrapped in growing peripheral nerves. It contains a strain sensor and a viscoplastic electrode composed of PEDOT:PSS as a conductor, and PDMS‐IU and PDMS‐IU_0.6_‐MPU_0.4_ as an insulator. b) Image of the 3D µCT scans of the sciatic nerve wrapped conformally by MorphE after chronic implantation. Scale bar: 200 µm. c) Stimulation (200 µs electrical stimulation pulse with 300 mV amplitude) by MorphE and cuff electrode, which induce compound action potential (CAP) for 8 weeks (left). Conduction velocity graph with chronic implantation showing that the cuff electrode was not measurable after 2 weeks regardless of whether the value of MorphE increased for 8 weeks (right). a–c) Reproduced with permission.^[^ ^50^ ^]^ Copyright 2020, Springer Nature. d) Schematic illustration of printing of a flexible silicon microdie. e) Photograph of >32 000 microdies on flexible substrates similar to the size of a human brain. f) Graph of time versus leakage current of thermally grown silicon oxide encapsulation on NMOS in 96 °C phosphate‐buffered saline (PBS). d–f) Reproduced with permission.^[^ ^53^ ^]^ Copyright 2019, The Authors, published by National Academy of Sciences, USA.

As rodents mature, the peripheral nerve also increases in diameter. Thus, the nerve cuff for recording peripheral nerve signals should have stretchability according to the growth rate of the nerve for long‐term recording. **Figure** **4**a shows a self‐healable, stretchable nerve‐cuff device using viscoplastic conductive polymers.^[^ ^50^ ^]^ The device measures the growth rate of neurons using a resistive strain sensor. To this end, the device was continuously stretched and contacted the nerve. As shown in Figure 4b, morphing electronics (MorphE) completely enclosed the nerve when simulated using 3D micro CT with negligible damage to it. Figure 4c shows that the conventional cuff electrode did not generate a meaningful biological signal when the peripheral nerves are stimulated from 2 weeks, whereas MorphE continuously measured and stimulated the sciatic nerve for ≈8 weeks. The prevailing problems for the existing cuff electrode array are that they are not stretchable to contain hazards of damages to the sciatic nerve and are detached after the diameter of the nerve is increased by a factor of 1.4. MorphE consists of viscoplastic material poly(dimethylsiloxane) (PDMS)‐IU0.6‐MPU0.4 and poly(3,4‐ethylenedioxythiophene) polystyrene sulfonate (PEDOT:PSS)/glycerol‐based strain sensors that enabled the enlargement of the device depending on the growth of the sheath. Furthermore, MorphE was seamlessly and conformably interfaced with the sciatic nerve with self‐healing properties. It did not require winding the device around the nerve, suturing it, and applying biocompatible glue for self‐healing. When examining the behavior of a rat with a conventional cuff electrode, it showed abnormal leg movements, whereas a rat implanted with MorphE showed normal gait. Sciatic nerve signal interrogation using viscoplastic electronic material helped the peripheral nerve to grow gradually, showing desirable compatibility with the surrounding nerves.

Likewise, for long‐term recording, the encapsulation layer of the device must not be degraded so that the layer blocks biofluids permeating into the device.^[^ ^51^ ^]^ Most polymer‐based encapsulation layers for implantable flexible electronics remain stable for several weeks.^[^ ^52^ ^]^ However, the layers are destroyed when pinhole defects occur during the process or in the biofluids. Delamination of the device may eventually occur months or years after surgery. Defect‐free thermally grown SiO_2_ (t‐SiO_2_) can be used as an encapsulation layer for measurement for at least 6 years. A large‐area flexible electronic/optoelectronic device was developed through transfer printing.^[^ ^53^ ^]^ Figure 4d shows how to produce scalable flexible electronics by fabricating a microdie on a wafer and repeating the transfer printing several times, then using PDMS for transfer to a large ​​flexible polymeric substrate. Figure 4e shows more than 32 000 microdies covering the area of ​​the human brain (≈150 cm^2^), obtained using this fabrication method. The primary sensory cortex part with touch, vision, auditory, and olfactory functions was transferred denser than the motor parts that control voluntary movements. Further, after transferring the microdies to t‐SiO_2_, the encapsulating microdie patterns with t‐SiO_2_ ensured that both sides are shielded with t‐SiO_2_. This structure demonstrated a significantly minimized leakage current measured for 9 days in phosphate buffered saline (PBS) at 96 °C, as shown in Figure 4f. This indicates that the leakage current has a reduced value with Arrhenius scaling for at least 6 years, and the device performance under a moisture environment confirms the capability of implanting the neural device chronically.

### 4.2 Bioresorbable Electronics

Bioresorbable electronics have been proposed as a solution to the problems of physical burden and financial loss by patients due to the secondary surgery required as a result of using non‐bioresorbable electronics.^[^ ^54^ ^]^ Bioresorbable materials decompose through continuous metabolic and hydrolytic reactions without causing problems in the body.^[^ ^55^ ^]^ The byproducts of the device are absorbed into the body or released out of the body. Through bioelectronics such as energy devices, sensors, and actuators made of these bioresorbable materials, the burden on the patient is eliminated by mitigating the problems that occur when a device is implanted into the body. In the following sections, various bioresorbable sensors and actuators are presented.

#### 4.2.1 Electrochemical Sensors

**Figure 5:**
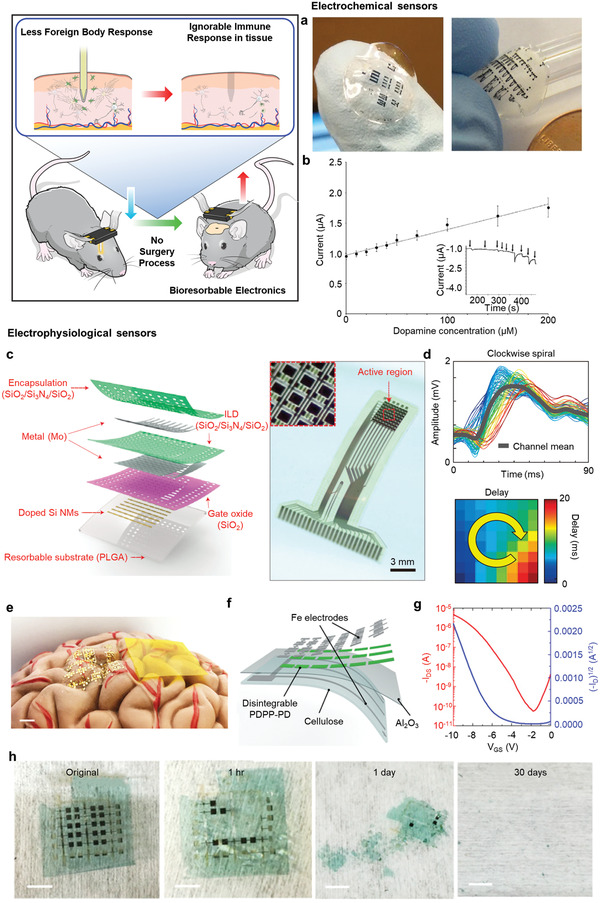
Expected advantage of in vivo reaction of bioresorbable material, no secondary surgery required. a) Silk fibroin sheet based bioresorbable electrochemical sensor for measuring dopamine (DA) and ascorbic acid (AA) concentration. b) Amperometric response curve of dopamine. Inset graph shows that current decreases with time. a,b) Reproduced with permission.^[^ ^57^ ^]^ Copyright 2016, Elsevier B.V. c) Schematic illustration of a bioresorbable active device for measuring electrophysiological signal (left). Photograph of a complete ECoG sensor (right). d) Data recording of epileptiform activity in a clockwise spiral form induced by picrotoxin. Recorded signal across the 64‐channels of the bioresorbable active device (top). Delay map for the bandpass filtered data of each recorded signal of 64‐channels (bottom). c,d) Reproduced with permission.^[^ ^59^ ^]^ Copyright 2016, Springer Nature. e) Pseudo‐CMOS array with biocompatible and totally disintegrable PDPP‐PD. Transferred onto a human brain model. Scale bar: 5 mm. f) Schematic illustration of totally disintegrable electronics with PDPP‐PD and iron electrodes. g) Transfer characteristics of totally disintegrable electronics. h) Images of dissolution of a totally disintegrable device with immersion in pH 4.6 buffer solution over time. Scale bar: 5 mm. e–h) Reproduced with permission.^[^ ^60^ ^]^ Copyright 2017, The Authors, published by National Academy of Sciences, USA.

Electrochemical sensing plays an important role in healthcare because the muscles, bones, blood vessels, and brain are affected by changes in the internal electrochemical properties of substances such as hormones and organic compounds. For instance, ascorbic acid (AA, vitamin C) and dopamine (DA) are in charge of forming collagen and transmitting nerve signals that are involved in emotional control.^[^ ^56^ ^]^ **Figure** **5**a shows a silk protein‐based electrochemical sensor that monitors dopamine and ascorbic acid concentrations.^[^ ^57^ ^]^ A conductive polymer, which serves as an active material instead of metals, silicon, or metal oxide, is mainly used as a component of the device. When manufacturing the device, sericin protein photoresist (SPP)‐PEDOT:PSS, a photoreactive conductive sensing ink, was used for water‐based photolithography, and the biodegradable sensor was completed by depositing it on a flexible silk protein fibroin substrate. Measurement was conducted through dopamine oxidation with a chronoamperometric setup at 0.3 V constant potential. The measurement result shows that the current increases with the increase in the dopamine concentration, as shown in Figure 5b. The above fully degradable electrochemical sensor is completely degradable by enzymatic action and has been active and stable for ≈1 month.

#### 4.2.2 Electrophysiological Sensors

It is possible to diagnose and treat neurological diseases by monitoring several electrophysiological signals in the body. Among them, voltage signal mapping of the brain plays an important role in identifying fundamental mechanisms and pathologies such as Parkinson's disease, epilepsy, and depression.^[^ ^58^ ^]^ Figure 5c shows an active device using highly doped Si nanomemebrane (NM) electrodes and Mo on a flexible polylactic‐*co*‐glycolic acid (PLGA) substrate.^[^ ^59^ ^]^ A bioresorbable ECoG monitors the electrophysiological signal on the cortical surface and the subgleal space. Mo was used as source, drain, gate electrode, and sensing electrode pads, SiO_2_ was used as gate dielectrics, and a tri‐layer of SiO_2_/Si_3_N_4_/SiO_2_ was used as interconnects, interlayer dielectrics, and blanket encapsulating layer. The electrodes, except the sensing sites, were separated from the biofluid and surrounding tissue. Figure 5d shows the results of measuring the induced spikes as a clockwise spiral when picrotoxin‐induced epileptiform activity occurs in the left hemisphere of rats through the 64 channels of the device. The structure of the pseudo‐complementary metal oxide semiconductor (CMOS) array using biocompatible and totally disintegrable polydiketopyrrolopyrrole‐p‐phenylenediamine (PDPP‐PD) is also shown in Figure 5e.^[^ ^60^ ^]^ Conformal contact with the brain model is shown, describing the ultraflexible and well‐isolated structure of CMOS. Figure 5f shows the manufacture of totally disintegrable electronics using PDPP‐PD and Fe electrodes. It was confirmed through the transfer characteristics that the transistor performance is well maintained while having bioresorbable characteristics, as shown in Figure 5g. Figure 5h shows the device disintegration performance over time, and it becomes totally disintegrable after 30 days. Both devices have features that enable the water‐aided peel‐off process of organic solvents by utilizing a regenerated cellulose substrate with high thermal stability, high transparency, and biodegradability.

#### 4.2.3 Pressure Sensors

**Figure 6:**
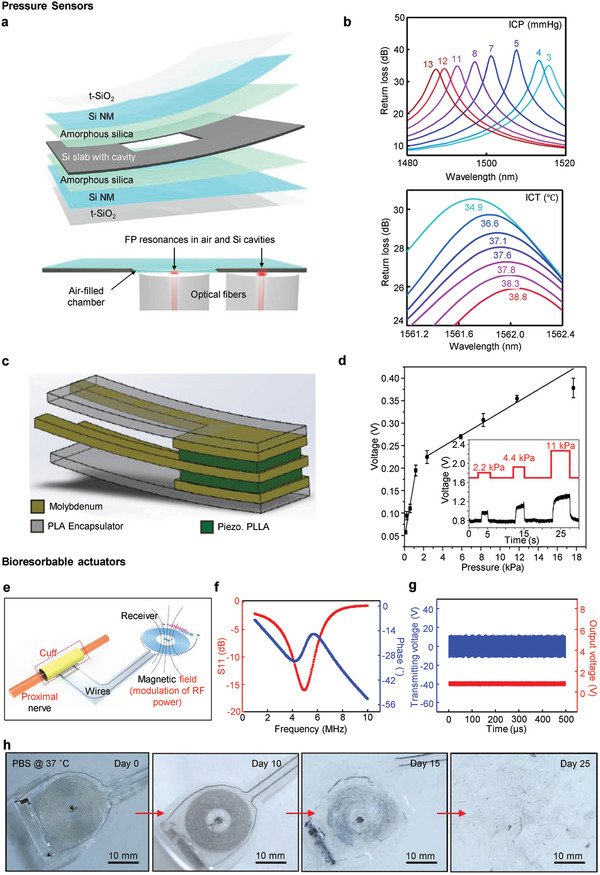
a) Schematic illustration of bioresorbable FPI pressure and temperature sensor design. b) Pressure calibration curve of an FPI sensor using data from ICP monitor (top). Temperature calibration curve of FPI sensor using data from ICT monitor (bottom). a,b) Reproduced under the terms of the CC‐BY Creative Commons Attribution 4.0 International license (https://creativecommons.org/licenses/by/4.0).^[^ ^63^ ^]^ Copyright 2019, The Authors, published by American Association for the Advancement of Science (AAAS). c) Schematic illustration of a biodegradable piezoelectric force pressure sensor. d) Pressure calibration curve of a piezoelectric PLLA sensor that measures voltage changes according to the applied pressure. The inset graph shows the output voltage signal from different constant forces. c,d) Reproduced with permission.^[^ ^64^ ^]^ Copyright 2018, The Authors, published by National Academy of Sciences, USA. e) Schematic illustration of a bioresorbable, wireless, programmable electrical stimulation device design. f) Radio frequency behavior of a bioresorbable stimulator. g) Generated output waveforms are alternated to sine wave current by the transmission coil. h) Image of dissolution of a wireless stimulator immersed in PBS (pH = 7.4) at 37 °C over time. e–h) Reproduced with permission.^[^ ^66^ ^]^ Copyright 2018, The Authors, published by Springer Nature.

Pressure monitoring of the body organ space is generally used to diagnose risk in the impaired organ and to prevent further damage.^[^ ^61^ ^]^ Moreover, it can be used for mechanical stimulation for tissue regeneration. For instance, monitoring of intracranial pressure for illustration function is essential for the treatment of traumatic brain injury, and by measuring the intraocular pressure, we can prevent the pathogenesis of glaucoma disease that leads to loss of vision.^[^ ^62^ ^]^ **Figure** **6**a shows the bioresorbable Fabry–Pérot interferometer (FPI) sensor for monitoring intracranial pressure and temperature using Si NM.^[^ ^63^ ^]^ An Si slab is used for the cavity as an optical sensor connected to the optical fiber and for the adhesion layer using amorphous silica. To prevent penetration of water from the bottom side, thermally grown silicon dioxide (t‐SiO_2_) is used for the encapsulation layer. As pressure is applied to the device, the air volume inside the silicon cavity chamber is deformed. With this deformation, intracranial pressure (ICP) calibration was performed by measuring the changes in the resonance peak of the optical spectra applied from the optical fiber. Similarly, the intracranial temperature (ICT) was calibrated by measuring the changes in the air cavity according to changes in temperature, as shown in Figure 6b. Figure 6c shows a pressure sensor using biodegradable piezoelectric poly‐l‐lactic acid (PLLA).^[^ ^64^ ^]^ The above device was constructed using polylactic acid (PLA) as the substrate and encapsulation layer, molybdenum as the metal lining, and placing piezoelectric PLLA at the center in the form of a sandwich. The device is composed of a multilayer with a sandwich‐type structure to achieve a higher piezoelectricity, enabling monitoring of various biological pressures. As shown in Figure 6d, we can confirm the piezoelectric characteristic that the flowing voltage increases as the pressure applied to the device increases.

#### 4.2.4 Bioresorbable Actuators

Actuators apply physical stimuli to the target organ through a variety of methods. These methods change the neuronal or chemical characteristic of the organ to treat abnormal functions. For instance, serious problems in healthcare such as nerve injuries inducing thermal stimulation with an inductive coil, pharmacological approach using drug delivery, and direct intra‐operative electrical stimulation.^[^ ^65^ ^]^ Figure 6e shows an implantable wireless electrical actuator utilizing an inductive coil.^[^ ^66^ ^]^ The device is designed to apply a certain amount of current to the target nerve by converting the power generated through radiofrequency (RF) waves. By employing the magnetic field applied to the inductive coil, the energy is transferred to the nerve cuff electrode through the wire. The inductive coil was designed with an RF diode using an Mg antenna and Si nanomembrane. A capacitor with an Mg/SiO_2_/Mg structure was designed for energy harvesting. A flexible, bioresorbable film made of PLGA was used as a substrate and encapsulation layer. Figure 6f,g shows the RF behavior of the stimulator and the mono‐phase output 1 V generated using the harvester. The device was inserted into the sciatic nerve of the rodent model to confirm regeneration through electrical stimulation. Figure 6h shows that the device is completely bioresorbed after 25 days.

### 4.3 Actively Multiplexed Electrode Arrays

Unlike the limitation of a passive electrode for high‐density and large area recording as it needs one wire per one electrode, an active neural electrode array can record neural signals with high resolution and higher number of electrodes since it includes transistors typically consisting of an amplifier and a multiplexer, which reduce the number of interconnection lines by connecting one wire per several electrodes.^[^ ^13^, ^32^, ^59^, ^67^ ^]^ Each column and row are connected with one line and can be controlled using multiplexing transistors. With this advantage, high‐density, high‐spatial resolution with high number of electrodes can be implemented on recording a larger area of neural tissues with high‐frequency band spiking activities, wave oscillation, and microseizures.^[^ ^32b^ ^]^ When applied to the surface of the heart, electrocardiogram (ECG) signals can be measured for each part of the heart. In the case of the brain, nerve signal propagation can be measured from a larger area of the target cells at the same time. The representative transistors used as a flexible, actively multiplexed electrode array include organic electrochemical transistors (OECTs), graphene‐based transistors, and transistors with an active layer of silicon.

#### 4.3.1 Organic Electrochemical Transistors (OECTs)

**Figure 7:**
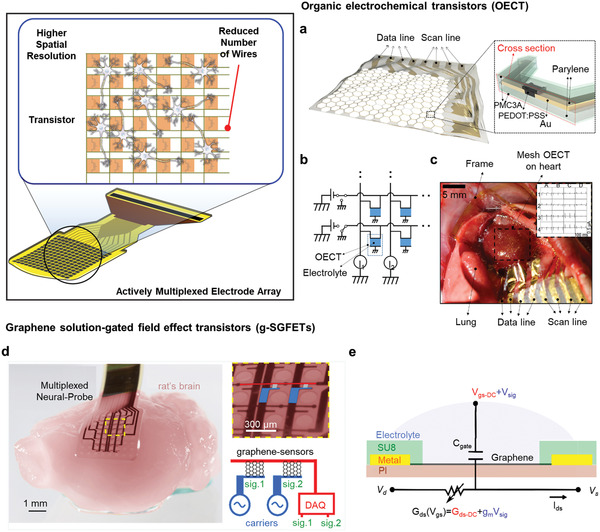
Expected advantages from active multiplex electrode array. a) Schematic illustration of a stretchable OECT active array with honeycomb grid pattern. The inset shows the cross view of a stretchable OECT array. b) Circuit diagram of a 4 × 4 OECT active array. c) Photograph of a 4 × 4 OECT array on a rat's heart. Scale bar: 5 mm. Inset shows the stable electrocardiogram (ECG) signals multiplexed by a 4 × 4 OECT array. a–c) Reproduced with permission.^[^ ^67e^ ^]^ Copyright 2018, The Authors, published by American Association for the Advancement of Science (AAAS). Reprinted/adapted from ref. ^[^ ^67e^ ^]^ . © The Authors, some rights reserved; exclusive licensee American Association for the Advancement of Science. Distributed under a Creative Commons Attribution NonCommercial License 4.0 (CC BY‐NC) http://creativecommons.org/licenses/by-nc/4.0/. d) Image of a graphene multiplexed active sensor array on a rat's brain. The inset shows a photograph of graphene sensor arrays. The illustration shows the mechanism of graphene sensors using frequency‐division multiplexing in which carrier signals are received from each channel and signals are received by the DAQ through graphene sensors that finally demodulates the carrier signals. e) Illustration of the equivalent circuit of graphene solution‐gated field‐effect‐transistors (g‐SGFETs). d,e) Reproduced with permission.^[^ ^67b^ ^]^ Copyright 2020, American Chemical Society.

OECTs have active organic components such as PEDOT:PSS as a channel and gold as an interconnect.^[^ ^67^, ^68^ ^]^ These elements have a superior transconductance of 1 mS, which is 100 times higher than that of a silicon field effect transistor.^[^ ^69^ ^]^ **Figure** **7**a shows OECTs on a flexible 1.2 µm thick parylene substrate with a honeycomb grid pattern. A 4 × 4 OECT array is stretchable, with a 1.2 µm thick parylene top encapsulation layer. Furthermore, poly (3‐methoxypropyl acrylate) (PMC3A) with non‐thrombogenic properties was fully encapsulated within a coat of 100–120 nm thickness to prevent the device from being degraded by blood generated by friction between the device and tissue. PMC3A has an excellent ionic conductivity even if fully encapsulated, and PEDOT:PSS can be operated as a gate channel. Blood does not stick, resulting in a continuous high signal‐to‐noise ratio (SNR) (≈52 dB) during measurement. Figure 7b illustrates the circuit diagram of a 4 × 4 OECT array. Each data line shows the desired point detected by applying an alternative voltage or 0 V, and all other lines are connected to the ground of the circuit to prevent crosstalk. When the heart is beating, the device can be stretched with a high SNR of 52 dB and its non‐thrombogenic property is not disturbed by bleeding caused by friction between the device and the tissue due to the heartbeat. High SNR ECG signals from a non‐thrombogenic, flexible, stretchable, and multiplexed 4 × 4 array were successfully measured with spatiotemporal resolution in the right and left ventricle areas of the rabbit heart, as shown Figure 7c.

#### 4.3.2 Graphene Solution‐Gated Field‐Effect Transistors (g‐SGFETs)

Two transistors are typically included in the active matrix for amplifying and multiplexing, respectively. However, a graphene solution‐gated field‐effect transistor (g‐SGFET) array uses one transistor with the frequency‐division multiplexing (FDM) method, which can reduce the needed interconnection lines compared with other neural implants, as depicted in Figure 7d,e.^[^ ^67b^ ^]^ Therefore, the device shows a flexible 4 × 8 active matrix capable of multiplexing without a switch. g‐SGFETs are graphene‐channel transistors that include a gate electrolyte, which is in contact with the brain tissue in in vivo experiments. The gate capacitance generated at the interface between the biological tissue and the device works as a sensing element, affecting the graphene channel. By fabricating g‐SGFETs as an array and receiving multiple carrier signals through FDM in one row line, the signals of each channel are distinguished by demodulating mixed signals using a lock‐in amplifier in a data acquisition system (DAQ). Demodulation of the FDM mode signal consisting of a lock‐in amplification scheme removes the noise of the amplifier, exhibiting a high mean sensitivity of 6.29 μV in the infra‐slow frequency band. With this switchless FDM method, the required interconnect line was reduced to 1 line per 1 column/row line in a total of 12 lines in a 4 × 8 active matrix, whereas the passive array requires 32 interconnect lines for 32 channels. Furthermore, by harnessing the material properties of graphene, it is possible to measure full‐band neural signals that can be acquired from a wider frequency band with infra‐slow band to high‐frequency components.

#### 4.3.3 Silicon Transistors

**Figure 8:**
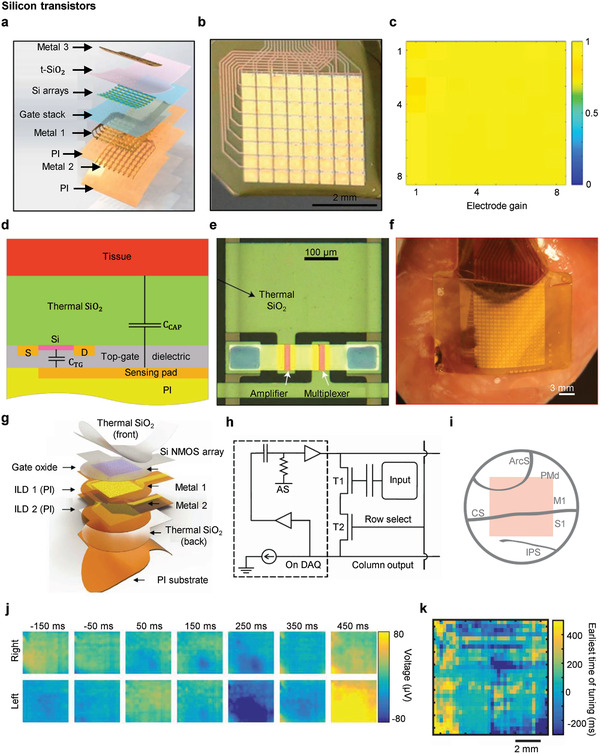
Silicon‐based transistor active array. a) Exploded view schematic illustrations and b) optical image of conductively coupled flexible active arrays. c) Spatial map of 64 channel active arrays with 100% yield. a–c) Reproduced with permission.^[^ ^67d^ ^]^ Copyright 2018, The Authors, published by National Academy of Sciences, USA. d) Circuit schematic of capacitively coupled arrays in cross‐sectional view. e) Photograph of a unit cell in the capacitively coupled array. f) Optical image of flexible, capacitively coupled arrays that conformably make contact with a rabbit's heart. d–f) Reproduced with permission.^[^ ^67c^ ^]^ Copyright 2017, Springer Nature. g) Exploded view schematic illustrations of “Neural Matrix” device, including ultrathin thermally grown silicon dioxide layers at the top and bottom sides for long‐term recording. It contains 1008 channels with capacitively coupled active arrays in which the unit cell includes two flexible silicon transistors. h) Circuit diagram of a single unit cell with amplifying and multiplexing transistors with an active shielding circuit on the DAQ. i) Schematic illustration of placement of an electrode array, shaded orange area on the cortical surface of non‐human primates. ArcS, arcuate sulcus. CS, central sulcus. IPS, intraparietal sulcus. PMd, dorsal premotor cortex. M1, primary motor cortex. S1, primary sensory cortex. j) Spatial map of motor evoked potentials array with the direction of the arm movement of non‐human primates. Signals of right movement for the top row and left movement for the bottom row. k) Spatial map of temporal neuronal signal propagation by “Neural Matrix” on a non‐human primate brain. g–k) Reproduced with permission.^[^ ^67a^ ^]^ Copyright 2020, The Authors, published by American Association for the Advancement of Science (AAAS).

It was mentioned above that t‐SiO_2_ can serve as the chronic encapsulation layer for neural implants; therefore, an active electrode array with silicon transistors has been developed using this stable silicon‐oxide layer. **Figure** **8**a shows an active matrix encapsulated in thermally grown SiO_2_ on an Si N‐channel metal oxide semiconductor (NMOS) array.^[^ ^67d^ ^]^ Figure 8b shows a 64‐channel 8 × 8 active matrix, with a conductively coupled active array that includes multiple transistors and amplifiers, two transistors per unit cell. The amplifier is connected to the gate electrode via conductive sensing through the p^++^Si//t‐SiO_2_ of the Au pad. Figure 8c shows a spatial map of the 64‐channel conductively coupled electrode gains with 100% yield. The deposition Au pad, after flipping using a flexible polyimide film and opening via t‐SiO2, creates a dual‐sided geometry that allows for a high‐contact area between electrodes and biotissues. P^++^Si works as an encapsulation layer with t‐SiO_2_ that rarely dissolves in biofluids and is free of defect formation during manufacture. When encapsulated with p^++^Si and t‐SiO_2_, PBS soaking test at 96 °C yielded stable results for 30 h, demonstrating its stability for 285 days with Arrhenius scaling. Furthermore, it interfaces conductively with biotissues on one side and channels of the silicon transistor on the other side for sensing and multiplexing. Using a conductively coupled 8 × 8 active matrix with dual‐sided geometry, neural signal interrogations are possible for a long period of time, which is helpful for superior spatiotemporal resolution.

Likewise, flexible active electronics have significant problems in terms of leakage currents due to the penetration of biological fluids or severe damage from direct metal contact with a tissue.^[^ ^67c^ ^]^ Figure 8d shows a device that measures ECG without direct contact through capacitive coupling in combination with t‐SiO2. t‐SiO2 was deposited on a Si NM transistor array as a complete leakage‐free encapsulation material. In Figure 8e,f, t‐SiO2 acts not only as an encapsulation layer, but also as an insulator inducing capacitive coupling between tissue and metal gates. Using this method, a low‐leakage current was maintained for 10 days at 10–9 A cm^−2^ for 120 days owing to the excellent encapsulation performance of t‐SiO_2_. The total thickness is ≈38 µm, ensuring flexibility and conformal contact. Figure 8f shows that ECG provided stable measurements when implanted into the rabbit's heart.

With increased number of channels of the above device, the high‐density active matrix of the micro‐electrocorticogram (μECoG), “NeuroMatrix,” was also demonstrated.^[^ ^67a^ ^]^ The device contains 1008 electrodes that are capacitively coupled, scaled‐up in a total area of ≈1 cm^2^, and with less than 100 interconnect wires by taking advantage of the active device. It was successfully inserted into non‐human primates to record brain signals in behavioral experiments. In Figure 8g, the exploded view of the entire device shows the total thickness of the existing capacitive circuit, ≈30 µm with high flexibility, allowing for conformal contact with the surface of the brain. Furthermore, it is expected to have an excellent encapsulation performance by using t‐SiO2. It was confirmed that it causes no problems in the brain tissue or device as a result of leakage current. Moreover, the output of the source‐follower amplifier is buffered in the existing capacitive coupled circuit, as shown in Figure 8h. The buffered output is an alternating current (AC) coupled to the direct current (DC) potential, connected to the drain of the source‐follower transistor. The transistors in an implanted area, the premotor, primary motor, and primary sensory cortices of the brain of a non‐human primate exhibited the spatiotemporal propagation of the neural signals by behavioral movements, as shown in Figure 8i. Figure 8j shows a spatial map of neural signals induced by the left and right arm movement directions. Figure 8k shows a propagation map of neural signals from the premotor cortex to the primary motor and sensory cortices with high spatiotemporal resolution.

### 4.4 Transparent Electrode Arrays

Optical image blocking and light‐induced artefacts during electrophysiological recording can be dramatically improved by using a transparent electrode array. The two main methods for fabricating transparent devices are: i) utilizing intrinsically transparent materials and ii) designing a special structure that ensures transparency. The first method involves designing an electrode array and substrate with materials that have high transmittance at the range of visible and near‐infrared wavelength. The second method involves using originally opaque metals that are visually transparent through mesh, grid, and nanowire (NW) structures. In the following sub‐section, simultaneous neural recording and imaging using graphene, CNT, and conductive polymer PEDOT:PSS‐based electrode array on a flexible poly(ethylene terephthalate) (PET) or PDMS substrate is discussed. The following section presents a structurally modified metal electrode array for electrophysiological readout with related studies.

#### 4.4.1 Utilizing Intrinsically Transparent Materials

**Figure 9:**
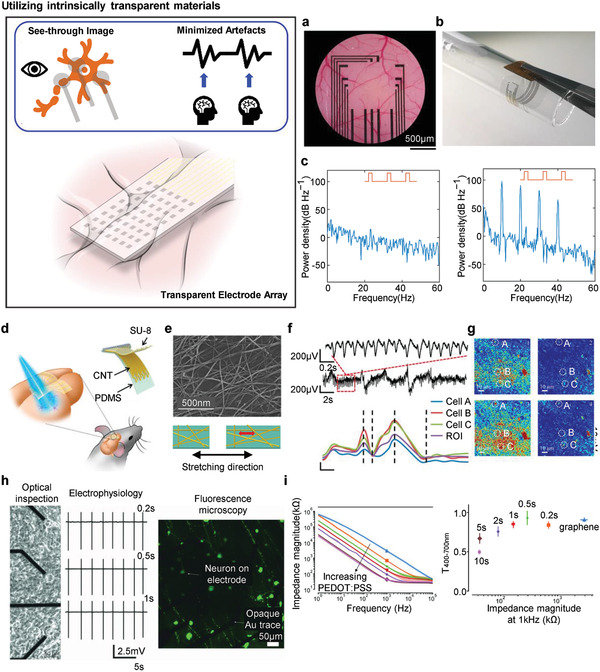
Utilizing intrinsically transparent materials. a) Optical image of transparent graphene micro‐electrode array (MEA) implanted on the surface of the brain. Scale bar: 500 µm. b) Photograph of a device wrapped on cylindrical surface (radius: 5 mm). c) Power density plot of a graphene electrode array (left) and Au control electrode array (right). a–c) Reproduced under the terms of the CC‐BY Creative Commons Attribution 4.0 International license (https://creativecommons.org/licenses/by/4.0).^[^ ^72^ ^]^ Copyright 2018, The Authors, published by Springer Nature. d) Schematic illustration of a stretchable, transparent carbon nanotube (CNT) electrode array. e) Scanning electron microscopy (SEM) image of CNT networks (top) and a schematic illustration of the stretched form (bottom). Scale bar: 500 nm. f) Ictal‐like discharges from the CNT electrode array (top) and Δ*F*/*F*_0_ traces of the brain under the recording site (bottom). g) Calcium images of the recording site during measurement of the ECoG. The four images correspond to the time points from the dashed lines in (f). The white dashed circles indicate the individual cells. d–g) Reproduced with permission.^[^ ^77^ ^]^ Copyright 2018, American Chemical Society. h) Optical image of a transparent graphene/PEDOT:PSS electrode array (left), electrophysiological recording (middle), and a fluorescence image of an implanted site with dyed neurons. Scale bar: 50 µm. i) Impedance plot of a bare and PEDOT:PSS‐coated graphene electrode array (left) and transmittance versus impedance plot of bare and PEDOT:PSS‐coated graphene electrode arrays at 1 kHz. h,i) Reproduced with permission.^[^ ^80^ ^]^ Copyright 2019, Wiley‐VCH.

Graphene is a 2D carbon‐based material, which is the most promising candidate for a transparent neural interface owing to its monoatomic structure.^[^ ^70^ ^]^ In addition to its desirable biocompatibility, graphene has high electrical conductivity and transparency of more than 90% in ultraviolet (UV) to infrared (IR) light. Transparent electrodes to interrogate biological dynamics require high transmittance to minimize light loss in the UV spectrum for optogenetic stimulation and in the infrared spectrum for photoinduced imaging.^[^ ^71^ ^]^ In this respect, graphene is advantageous for optical imaging because it exhibits gradient roll‐off in IR in contrast to indium tin oxide (ITO) and ultrathin metal film.^[^ ^36^ ^]^ Utilizing its unique properties, a 1200 µm deep two‐photon imaging and electrical response from optogenetic stimulation were successfully obtained with a graphene electrode.^[^ ^72^ ^]^ **Figure** **9**a shows the see‐through image of the somatosensory cortex of a mouse using a graphene electrode array on a PET substrate. Graphene has an extra spatial degree of freedom to maintain flexibility owing to the hexagonal bonding of the carbon atoms. Negligible impedance variation was observed when the array was wrapped around a cylindrical rod to demonstrate its reliability at a curved surface, as shown in Figure 9b. Light‐induced artefacts were also analyzed in vitro with both the graphene electrode and a control sample. Figure 9c shows the power density spectra of a 10 Hz light pulse illumination of graphene and gold electrode. The amplitude of the artefacts was significantly diminished by utilizing graphene at a low frequency, unlike gold electrodes that have four amplitude peaks within the specified frequency range.

Another example of using the flexible and transparent nature of a carbon‐based material is the use of CNTs. CNTs have been widely implemented on flexible substrates as an electrode array owing to its superior electrical conductance with large capacitance.^[^ ^73^ ^]^ CNTs can be coated on neural interface substrates, thereby ameliorating neuronal proliferation as capacitive electrochemical electrodes.^[^ ^74^ ^]^ They are divided into single‐walled carbon nanotubes (SWCNTs) and multi‐walled carbon nanotubes (MWCNTs) based on the number of graphene sheets.^[^ ^75^ ^]^ They have different material properties depending on their chirality. SWCNTs as a conducting material have much more flexibility than MWCNTs, with metallic properties when the carbon networks are in an armchair structure.^[^ ^76^ ^]^ A transparent CNT‐based electrode can be designed employing the intrinsically void area of the carbon network, while a low tube‐to‐tube contact resistance guarantees a desirable electrical conductivity. Figure 9d,e shows the schematic illustration and scanning electron microscopy (SEM) image of a stretchable CNT electrode array on a transparent PDMS substrate for simultaneous optical imaging with electrical recording.^[^ ^77^ ^]^ The web‐like form of the CNTs allows for omni‐directional stretchability and optical transparency (transmittance of more than 85% at a wavelength of 400 nm). The simultaneous calcium imaging and ECoG recording in Figure 9f,g show that CNTs are suitable candidates for a transparent electrode, causing minimal imaging artefact during electrophysiological interrogation.

Conductive polymers have a form in which the chain intersects a double bond with an unsaturated hydrocarbon and a single bond with a saturated hydrocarbon.^[^ ^78^ ^]^ PEDOT:PSS is one of the polymers with conductive property owing to delocalization of electron density by pi‐bonding. PEDOT:PSS is widely used in organic light emitting diodes (OLEDs) and solar cells because of its superior charge injection capacity and high light transmittance.^[^ ^79^ ^]^ Because of the similarity of its modulus with that of a tissue as well as its biocompatibility, PEDOT:PSS is also applied to implantable electrode coating, modulating its crystallinity, as shown in Figure 9h.^[^ ^80^ ^]^ In combination with a monolayer graphene, PEDOT:PSS improved the electrochemical impedance, while maintaining its transparency, as shown in Figure 9i.

#### 4.4.2 Designing a Special Structure to Ensure Transparency

**Figure 10:**
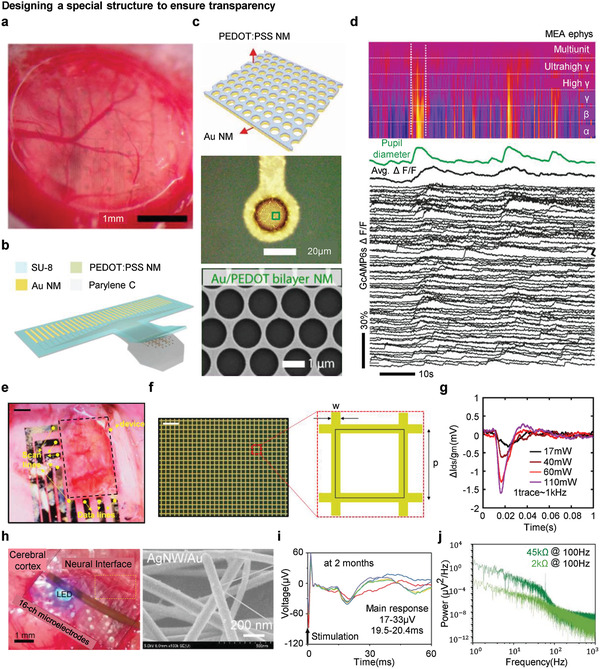
Designing a special structure to ensure transparency. a) Optical image of a transparent MEA closed with the cranial window, maintaining its transparency. Scale bar: 1 mm. b) Schematic illustration of the device. Au/PEDOT:PSS nanomesh structured electrode array is deposited on a parylene C substrate and encapsulated with a thin layer of SU‐8. c) Schematic illustration of a bilayer nanoscale electrode pattern (top). Optical microscopy image of a single‐channel Au/PEDOT:PSS nanomesh microelectrode (middle). SEM image of the Au/PEDOT:PSS bilayer nanomesh from the green‐dashed line in the image above (bottom). d) Simultaneous electrophysiological recording (spectrogram), arousal (pupil diameter), and two‐photon imaging (Δ*F*/*F* traces representing a single‐neuron Ca++ activity). a–d) Reproduced with permission.^[^ ^81^ ^]^ Copyright 2018, The Authors, published by American Association for the Advancement of Science (AAAS). Reprinted/adapted from ref. ^[^ ^81^ ^]^. © The Authors, some rights reserved; exclusive licensee American Association for the Advancement of Science. Distributed under a Creative Commons Attribution NonCommercial License 4.0 (CC BY‐NC) http://creativecommons.org/licenses/by-nc/4.0/. e) Transparent organic electrochemical transistor (OECT) array consisting of an Au grid network‐based interconnection with a PEDOT:PSS active layer. Scale bar: 1 mm. f) Optical image of the Au grid on a parylene substrate (thickness: 1.2 µm). Scale bar: 100 µm. A magnified image of a metal grid and square grid unit cell parameters. g) Electrophysiological signal by transparent OECT with a 475 nm wavelength optical stimulation according to the light intensity. e–g) Reproduced with permission.^[^ ^68b^ ^]^ Copyright 2017, The Authors, published by National Academy of Sciences, USA. h) Image of a transparent electrode array based on an AgNW/Au network with a light emitting diode (LED) implanted on the cerebral cortex. Scale bar: 1 mm (left), and SEM image of the AgNW/Au network at the tracks of an electrode array. Scale bar: 200 nm (right). i) Electrical signal from the rat's brain with a tibial nerve stimulation over a 2 month implantation. j) Fast Fourier transform (FFT) power versus frequency plot for noise measurement from a neural interface. h–j) Reproduced with permission.^[^ ^82^ ^]^ Copyright 2019, The Authors, published by Wiley‐VCH.

Metal‐based materials for biointegrated electronics are originally opaque, hence they block the field of view of neural tissues while observing tissue dynamics with a microscope. Exploiting special structures is an attractive approach to enable an intrinsically opaque metal to be transparent. Because a trade‐off exists between transparency and conductivity, coating with a low impedance material, such as the conductive polymers shown in **Figure** **10**b, and optimizing the interface design, as shown in Figure 10f, were carefully considered. A PEDOT:PSS‐coated gold nanomesh (NM) transparent electrode was used for electrophysiological readout, as depicted in Figure 10a.^[^ ^81^ ^]^ The relatively high electrochemical impedance as a result of the small electrode size and NM structure was improved by coating the PEDOT:PSS nanomembrane, as shown in Figure 10c. An SEM image of the zoomed‐in region of the electrode shows a uniform structure of NM densely packed with many void spaces, resulting in a desirable light transmittance. Figure 10d depicts the simultaneous potential recording with two‐photon calcium imaging using the visual stimuli of a mouse that is awake. The correlation between biological spectra with undisturbed Ca^++^ traces clearly shows the compatibility of the transparent electrode array as a multimodal recording device.

Designing a metal grid structure is another well‐known approach for transparent electronics. Figure 10e shows transparent OECTs.^[^ ^68b^ ^]^ Intrinsically transparent PEDOT:PSS was used as an active layer, and an Au grid structure was applied to the source/drain, including the interconnection. A metal grid structure differs in transparency, and depends on the width (*w*) of the grid and the periodicity (*p*) of the unit, as shown in Figure 10f. In the following experiment, the optimal w and p parameters were observed to apply sufficient voltage from the contact pads to the transistors, with high transparency. This approach minimized the reflection of the laser stimulation with different excitation intensities, as demonstrated in Figure 10g.

Metallic NW structures have more distinctive properties than metal films with apertures. Metallic NWs have excellent electrical conductivity for high surface areas and an aspect ratio with optical transparency, making them attractive candidates for optoelectronics. Furthermore, wire–wire junctions have much higher mechanical flexibility than carbon and metal oxide‐based electrodes; therefore, they are advantageous for tissues with flexion and neural interfaces requiring direct contact. One of the considerations when utilizing metal NWs in implantable devices is electrode failure based on foreign body response due to wire oxidation. Figure 10h shows a neural interface using an Ag/Au NW core–shell.^[^ ^82^ ^]^ To overcome fast oxidation of the Ag NW, a transparent hydrogel was coated at the sensing site to achieve minimal light loss. Figure 10i,j shows the somatosensory‐evoked potentials after 2 months of implantation and noise measurement combined with optogenetics, respectively. The figure indicate that metal NWs are attractive alternatives to neuro‐prosthetics, reducing light‐induced artefacts by optical stimulation.

### 4.5 Wireless Electronics

Movement constraints from physically tethered, externally wired implants can be resolved by using integrated wireless systems.^[^ ^83^ ^]^ Here, we discuss wireless electronics for a neural interface from two perspectives: i) classification by the type of wireless communication and ii) actuation of device operation by wireless stimuli. The first approach includes the Bluetooth system, near‐field communication (NFC), and radiofrequency identification (RFID). The second approach mainly focuses on wireless mild‐thermic, light‐induced actuation, and ultrasonic wave. A brief overview of each wireless communication with the related research and studies on wirelessly triggered device operation is presented in the following sections.

#### 4.5.1 Classification by Type of Wireless Communication

**Figure 11:**
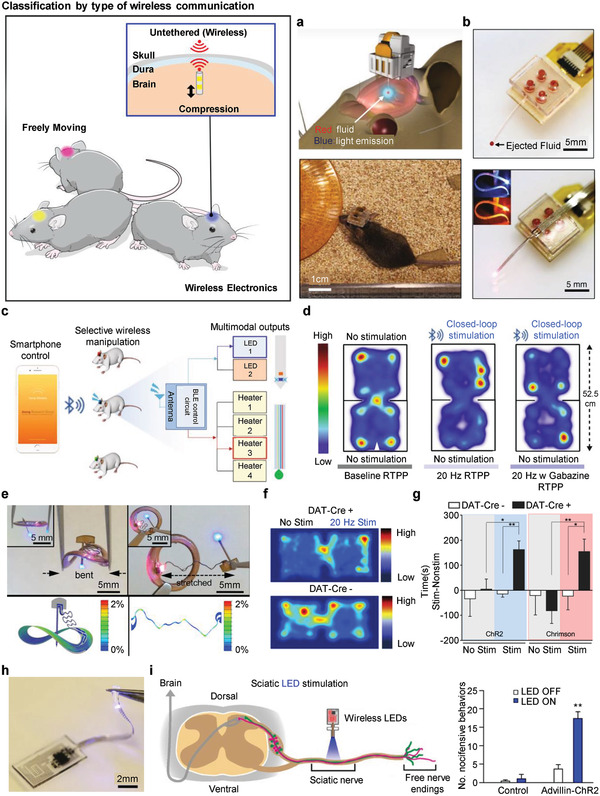
Classification by type of wireless communication. a) Schematic illustration of simultaneous optical/chemical modulation of a brain with an implantable device integrated with a Bluetooth system (top) and an implanted mouse (bottom). b) Microfluidic function of the device (top) and a photograph of the capability of optical stimulation (bottom). c) Smartphone Bluetooth system for wireless manipulation of a specific mouse in a group of multiple mice implanted with an optofluidic device. d) Heatmap depicting mice locomotion from 20 min RTPP sessions with baseline (left), 20 Hz photostimulation (middle), and 20 Hz photostimulation with an injection of gabazine (right). The area is divided into two sections, namely stimulation site and non‐stimulation site. a–d) Reproduced with permission.^[^ ^84^ ^]^ Copyright 2019, The Authors, published by Springer Nature. e) Photographs of near field communication (NFC)‐based wireless implant and device modeling results before and after bending the body of an implant (left) and stretching the injectable needle (right), respectively. f) Heatmap of locomotion of DAT‐Cre+ or DAT‐Cre‐ mice at differently conditioned chambers. g) Experimental results from chamber preference test from DAT‐Cre‐ and DAT‐Cre+ mice according to different injections. e–g) Reproduced with permission.^[^ ^85^ ^]^ Copyright 2016, Elsevier B.V. h) Photograph of a radio‐frequency (RF) powered implantable device in a connection with LEDs. Scale bar: 2 mm. i) Schematic illustration of nociceptive pathways and optical stimulation of the sciatic nerve (left) and the number of nocifensive behaviors from the control mice and Advillin‐ChR2 mice controlled using an LED stimulator (right). h,i) Reproduced with permission.^[^ ^86^ ^]^ Copyright 2015, Springer Nature.

The Bluetooth system is a bidirectional, master–slave technology working at a range of 2.4 GHz. It is a proprietary protocol, which requires pairing with smart electronics. An emerging Bluetooth system implemented on implantable devices is Bluetooth low energy (BLE). BLE's communicating mechanism is designed to be suitable to transmit intermittent short bursts, rather than continuous data streams applied in conventional consumer applications. This resulted in significant improvement in the power consumption issue in the existing Bluetooth. Research on flexible wireless probes for neuropharmacology and photostimulation through BLE is depicted in **Figure** **11**a,b.^[^ ^84^ ^]^ Wireless control of drug and light emission were demonstrated, with the capability for behavioral mouse experiments without any movement constraints. Figure 11c shows the schematic diagram of smartphone BLE control of a target mouse implanted with the device. By pairing with a device implanted in the cortex of the mouse, the user can selectively transmit Bluetooth signals in the vicinity of 10–100 m. Users can determine the operating conditions, such as the light emitting diode (LED) frequency or heater target number, from the device (slave) through the smartphone (master). Representative heatmaps of the real‐time place preference (RTPP) of mice with implanted devices were also obtained, as shown in Figure 11d. Photostimulation and drug (Gabazine, receptor antagonist) delivery through BLE demonstrate the predetermined locomotor activity of freely moving mice.

NFC is a bidirectional, local area network that operates in the 13.56 MHz band. The distance of data exchange is just a few centimeters, but the communication sequences are automatically performed when adjacent distances are reached without a separate pairing process. Furthermore, NFC takes up fewer footprints than BLEs, which can improve user convenience, and it is advantageous in terms of utilizing the battery space without built‐in power. The flexible NFC electronics for optogenetics are demonstrated in Figure 11e.^[^ ^85^ ^]^ The device can be bent or stretched through serpentine interconnects bypassing the strain, as obtained using finite element analysis (FEA). The device receives power through magnetic coupling with loop antennas at high frequency bands, thereby circumventing the susceptibility to signal reflection and interference. A heatmap of the RTPP test with mice expressing ChR2 and preference plot are shown in Figure 11f,g, respectively. As determined by previous research, an integrated wireless system in a chip is a suitable candidate for behavioral studies.

RFID is a wireless technology that uses radiofrequency, particularly for unidirectional communication, whereas the Bluetooth system and NFC are bidirectional. RFID is the same as NFC in that the signal is received from an RF harvester through externally applied electromagnetic waves. For the most part, a rectifier, such as Schottky diodes, is required for implantable device operation, replacing the delivered RF signals with DC sources. Figure 11h depicts a fully implantable wireless device for the optical modulation of mice.^[^ ^86^ ^]^ Capacitive coupling induced by the adjoining traces of the antenna lowers the resonant frequency, thereby miniaturizing the entire size of the device. A schematic illustration and experimental results of nociceptive pathways with wireless LED stimulation of the sciatic nerve are shown in Figure 11i.

#### 4.5.2 Actuation of Device Operation through Wireless Stimuli

**Figure 12:**
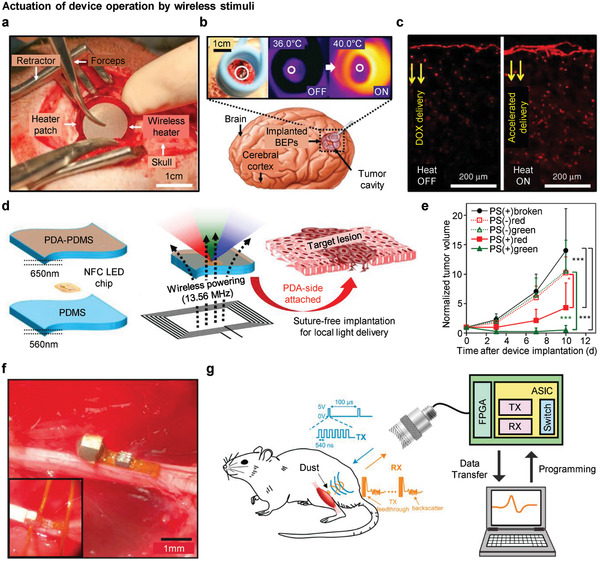
Actuation of device operation by wireless stimuli. a) Implantation of a wireless, thermally activated drug delivery device in the canine model. b) Schematic illustration of wireless mild‐thermic actuation of the device. c) Fluorescence images of doxorubicin (DOX) diffusion from the device into the J3T‐1 tissue without (left) and with (right) wireless thermic actuation. a–c) Reproduced under the terms of the CC‐BY Creative Commons Attribution 4.0 International license (https://creativecommons.org/licenses/by/4.0).^[^ ^87^ ^]^ Copyright 2019, The Authors, published by Springer Nature. d) Schematic illustration of a wireless, photoactuated cancer therapy device (left), working principle of a wirelessly powered system with target lesion (right). e) Normalized tumor volume of the three controls (PS(+)broken, PS(−)red, and PS(−)green) and two photodynamic therapy (PDT) groups (PS(+)red and PS(+)green) of the experimental mice. d,e) Reproduced with permission.^[^ ^88^ ^]^ Copyright 2019, The Authors, published by Springer Nature. f) Optical image of ultrasonically actuated neural dust mote implanted onto the sciatic nerve in a rat model. Scale bar: 1 mm. g) Experimental setup for EMG recording from gastrocnemius muscle in rats. f,g) Reproduced with permission.^[^ ^89^ ^]^ Copyright 2016, Elsevier B.V.

A wireless system that can treat abnormalities in biological conditions usually depends on external stimuli such as heat, light, and ultrasound. These resources are used to indirectly activate a target layer or directly implemented as a signal transmitter or cancer treatment. As an example of a wireless mild‐thermic activation of drug delivery, a biodegradable patch integrated with wireless electronics is shown in **Figure** **12**a.^[^ ^87^ ^]^ The device consists of a drug‐loaded layer, wireless heater, and temperature sensor. A magnetic field of 220 kHz, 360 A is applied to the heater, which acts as an accelerator of intracellular diffusion of the drug. Figure 12b shows an illustration of wirelessly controlled temperature changes at a distance between the coil and the heater. To prevent acute thermal damage to the brain, temperature transition was carefully measured using a wireless sensor. As shown in Figure 12c, accelerated drug delivery by magnetic‐field‐coupled thermic actuation was observed through fluorescence microscopy images.

The light source is another consideration for cancer therapy or tumor treatment. Photodynamic therapy (PDT) is a representative method, which is a technique of injecting photosensitive drugs in cancerous cells, thereby activating them with special wavelengths. Figure 12d depicts an exploded view of a wirelessly powered device for PDT with the schematic diagram of the implantation procedure.^[^ ^88^ ^]^ An NFC‐based LED chip is inserted in the flexible PDMS substrate. An LED encapsulation layer also contains a tissue‐adhesive substance, enabling stable contact with shear force or compression from the device migration. A normalized tumor volume was significantly decreased in two PDT groups (PS(+) red and PS(+) green) using electromagnetically coupled light source, as shown in Figure 12e.

Further miniaturization of the wireless device requires more pinpoint coupling with an external source, sustaining its reliability. Ultrasound (US) offers unique advantages for wirelessly powered implantable devices with the desirable spatial resolution from small wavelengths and high penetration depth in a tissue than electromagnetic radiation. In Figure 12f, neural dust operated by a wireless ultrasound source is implanted in the sciatic nerve.^[^ ^89^ ^]^ Ultrasonic energy is applied using an external transducer, following source conversion from US to current through piezocrystal vibrations. The current supplies power to the transistor, altering the gate through the electrophysiological potential difference between two electrodes. Current variations by the transistor in turn modulate the vibration of the piezocrystal. The reflected ultrasonic pulses from the piezocrystal indicate the encoded result of the biological signal. A detailed schematic illustration of the principle of neural dust is depicted in Figure 12g.

### 4.6 Integrated Recording and Stimulation Electronics

Since monofunction devices have only one function of recording or stimulation, they have limitations in providing appropriate treatment for patients. A multifunctional device that performs integrated recording and stimulation has multifunctional capability that can be exploited as therapeutic devices by monitoring health conditions and modulating neuronal dynamics.^[^ ^45^ ^]^ The requirement of such multifunctionality is to form a closed‐loop control (feedback system) for scrutinizing various disorders in real time in situ and to provide efficient modulation platforms corresponding to the biological information obtained from the body.^[^ ^90^ ^]^ For instance, epilepsy is an abnormal synchronization of the action potentials generated in the brain resulting in seizures or an unusual behavior. In this respect, the device must continuously maintain real‐time electrophysiological recording with efficient drug delivery or electrical stimuli.^[^ ^91^ ^]^ The time‐resolved feedback system offers improved clinical procedures in acute situations according to the conditions of patients. In the following sections, we discuss recent simultaneous recording and modulation devices with the integrated fabrication techniques of the device.

#### 4.6.1 ECG + Strain Gauge + pH + Temperature + Optical Sensor with Electrical/Optical Stimulation

**Figure 13:**
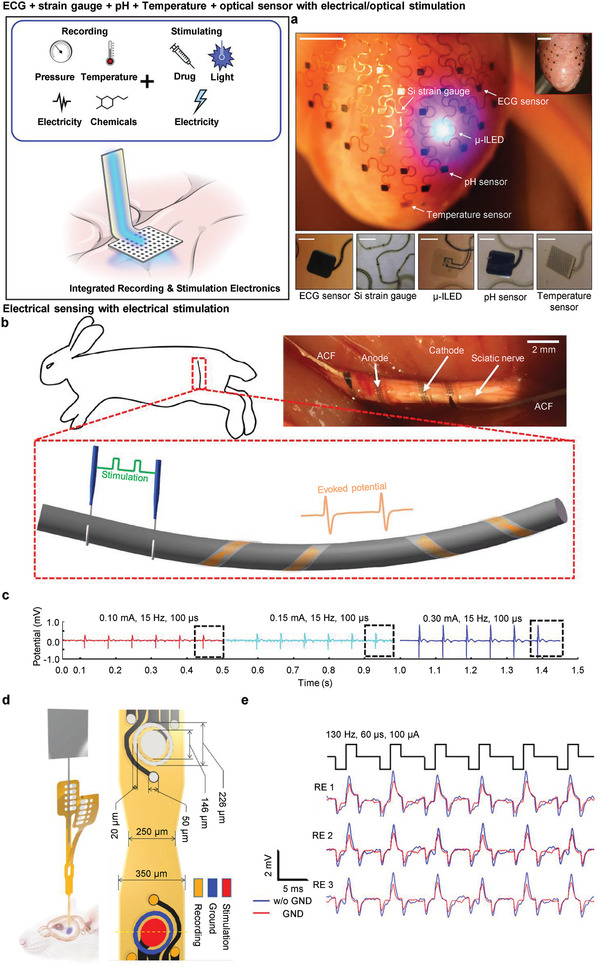
Expected advantages of integrating various stimulation and recording technologies. a) 3D multifunctional integumentary membranes (3D‐MINs) integrated on a rabbit's heart. Top scale bars: 6 mm. Bottom scale bars: 500 µm. Reproduced with permission.^[^ ^14^ ^]^ Copyright 2014, Springer Nature. b) Schematic illustration of self‐climbing twining electrodes using shape memory and in vivo experimental setup for electrical recording and stimulation on the sciatic nerve. c) Recorded CNAPs induced by the applied current in in vivo experiment. b,c) Reproduced under the terms of the CC‐BY Creative Commons Attribution 4.0 International license (https://creativecommons.org/licenses/by/4.0).^[^ ^95^ ^]^ Copyright 2019, The Authors, published by American Association for the Advancement of Science (AAAS). d) Schematic illustration of a flexible, multifunctional neural probe for electrical stimulation and recording. e) In vivo stimulation/recording data with and without GND. d,e) Reproduced with permission.^[^ ^96^ ^]^ Copyright 2018, Elsevier B.V.

Since the heart acts as a biological pump that supplies enough blood to all cells, the tissues and organs of the body must respond to a variety of metabolic demands from numerous working elements from a complex electromechanical syncytium.^[^ ^92^ ^]^ Therefore, mapping and stimulation of various physiological parameters while maintaining high‐spatiotemporal resolution play an important role in cardiology. In **Figure** **13**a, an integrated device with an ECG sensor, Si strain gauge, pH sensor, and temperature sensor are demonstrated.^[^ ^14^ ^]^ The device is used to perform various physiological signal mappings in the heart, which provide the route for signal propagations. Simultaneous optical mapping and stimulation were performed successfully using a combination of a monitoring sensor and a micro‐LED. To design each sensing/stimulation part of the device, indium gallium nitride (InGaN), silicon nanomembranes, gold electrode, and iridium oxide (IrOx) pads were used for optical mapping/stimulation, strain gauges, electrical sensing/stimulation, and pH sensing, respectively. Serpentine structures of resistors using gold yielded a desirable performance for temperature sensing/heaters. The device comprises 3D, multifunctional exterior membranes (3D‐MIMs) that can be manufactured in a custom‐made form using a 3D printer, enabling it to build a conformal interface at all points of the heart.

#### 4.6.2 Electrical Sensing with Electrical Stimulation

Electrophysiological monitoring and stimulation through electricity are the most common methods used for modulating neural dynamics. This electrical interface affects the ionic environment by measuring the action potential delivered by the electrogenic cell through the sensing pads or by injecting charge to the electrodes.^[^ ^93^ ^]^ However, in the case of electrical stimulation, it is difficult to selectively control activities in a target tissue and it serves as an impediment that prevents accurate measurement of the desired signals. Another disadvantage is that only neuronal firing is possible.^[^ ^94^ ^]^ Figure 13b shows a twining electrode wrapped around the peripheral nervous system implanted into the rabbit's sciatic nerve for simultaneous electrical monitoring and electrical stimulation.^[^ ^95^ ^]^ The peripheral nerve plays an important role in communication between the central nervous system and various motor/sensory neurons. Electrical stimulation has been proposed as a therapeutic strategy for neurodegenerative disorders from peripheral nerves. Au/polyimide (PI) with a meshed serpentine structure was employed by transferring it to shape memory polymers (SMPs) using a transfer printing process. SMPs respond to the body temperature; therefore, when they come in contact with the peripheral nerve, they self‐climb to make conformal contact with the target nerve. Because of these characteristics, the mechanical and geometrical mismatch that occurs during nerve integration with the existing 2D device is significantly improved. Figure 13c shows the compound nerve action potentials (CNAPs) measured while stimulating various currents in the nerve. CNAPs change with increasing current intensity, which confirms that recording and stimulation were well performed. Figure 13d shows a flexible deep brain neural probe for interrogating signals with adequate therapy.^[^ ^96^ ^]^ The device used a Ti/Pt layer as the stimulation electrode, a Ti/Au layer for recording, and a ground (GND) electrode on the polyimide substrate. There is a stimulation electrode in the center of the device in the form of a large circle and circular grounding surrounding the stimulation electrode. The problem that occurred during the electrical stimulation described above was resolved using grounding, so that the stimulation target point can be specified more precisely, and it was possible to conduct the measurements more accurately and reliably. Figure 13e shows the result of in vivo stimulation/recording; the biological data is more accurately displayed depending on the absence or integration of GND.

#### 4.6.3 Electrical Sensing with Optical Stimulation and Drug Delivery

**Figure 14:**
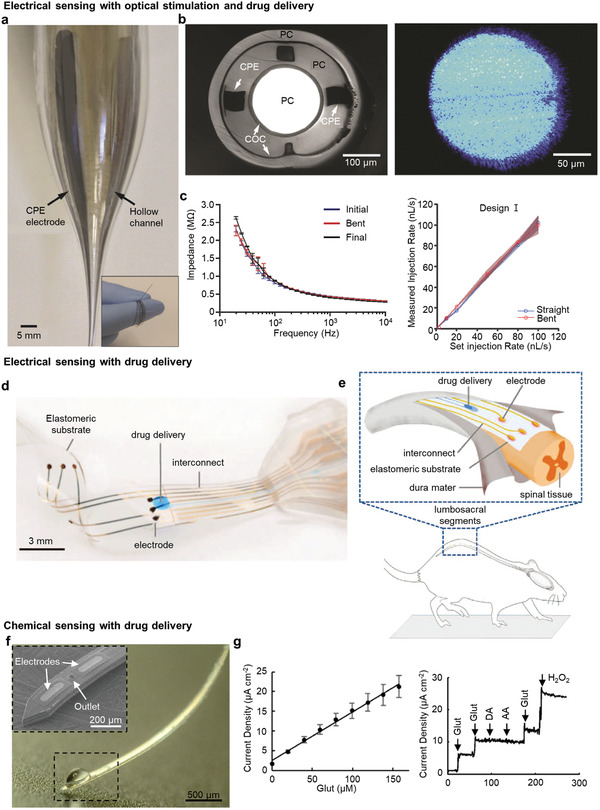
a) Photograph of the preform and drawn fiber produced by thermal drawing process (TDP). b) Cross‐sectional optical images of the multifunctional fiber probe (left). Cross‐sectional optical images of the light (right). c) Impedance spectroscopy of the multifunctional fiber probe in various fiber conditions (left). Injection rate of the multifunctional fiber probe (right). a–c) Reproduced with permission.^[^ ^99^ ^]^ Copyright 2015, Springer Nature. d) Photograph of the electronic dura mater for long‐term measuring electrical signals with chemical and electrical stimulation. e) Schematic illustration of the electronic dura mater integrated on the spinal subdural space of a rat. d,e) Reproduced with permission.^[^ ^102^ ^]^ Copyright 2015, American Association for the Advancement of Science (AAAS). f) Optical images of the multifunctional flexible probe for measuring electrochemical sensing with chemical stimulation. g) Calibration curve of the electrochemical sensing probe for measuring current density according to repetitive addition of glut (left). Recorded comparison graph of current density changes according to the addition of glut, dopamine (DA), and ascorbic acid (AA) (right). f,g) Reproduced with permission.^[^ ^103^ ^]^ Copyright 2019, Elsevier B.V.

Optogenetics have been thoroughly studied in neuroscience as an optical modulation method.^[^ ^97^ ^]^ The optogenetic method transforms the target cells through gene expression of a light‐sensitive ion channel (opsin), thereby delivering different wavelengths of light to excite or inhibit. This method has the advantage that it can be implemented by targeting a specific neural population. However, it still has limitations that prevent it from being applied to humans, in terms of the hazard of injecting a virus to change the light sensitivity of the target neuron.^[^ ^98^ ^]^ **Figure** **14**a shows a fiber‐based probe capable of simultaneous electrical monitoring/optical and chemical modulation.^[^ ^99^ ^]^ A thermal drawing process (TDP) was used for manufacturing a multifunctional probe, as shown in the figure. The TDP process exploits the general optical fiber production and has the advantage that the preform is scaled down as it is manufactured. Figure 14b shows a cross‐sectional view of the fabricated fiber. As shown in the exploded component of the device, a conductive fiber (CPE) was used for the electrical electrode, a polycarbonate (PC) was used for the optical waveguide, and a hollow channel was used for the microfluidic channel. The figure on the right shows that light is transmitted through the PC optical waveguide. Similarly, the graph in Figure 14c (left) shows that electrical monitoring can be performed without variations even when bent, and the injection rate of drug delivery is shown in the graph on the right.

#### 4.6.4 Electrical/Chemical Sensing with Drug Delivery

Chemical monitoring has the advantage of quickly detecting electroactive molecules directly, but it is prone to problems in terms of selectivity and biofouling. Chemical modulation has a great advantage as a neuromodulator and therapeutic agent because it can be delivered in situ directly to the target region.^[^ ^100^ ^]^ Through this delivery process, it is used to inject a therapeutic agent to alleviate the foreign body response as a result of device insertion, or to strengthen a specific neural behavior.^[^ ^101^ ^]^ Figure 14d shows an electronic dura mater that integrates drug delivery and electrical read/write electrodes.^[^ ^102^ ^]^ A device substrate and a microfluidic channel were fabricated for the above device using PDMS to minimize mechanical mismatch with the tissue. Figure 14e shows a device inserted into the spinal subdural space of a rat. An experiment was conducted through electrical stimulation pulse and chemical injection to restore the locomotion disorder caused by a paralyzing spinal cord injury. In the case of Figure 14f, the device is a flexible neural probe that combines chemical sensing and drug delivery.^[^ ^103^ ^]^ The device has a port for drug delivery and a site for electrochemical sensing through a PDMS thin‐film transfer process. For electrochemical sensing, glutamate (glut) was measured using a Pt microelectrode and a poly‐m‐phenylenediamine (PPD) layer was deposited on the Pt to detect DA and AA. The graph on the left side of Figure 14g shows that it is possible to confirm that the current density increases according to the concentration of the glut, and the adjacent graph (right) shows that the current density does not change when DA and AA are injected.

## 5 Conclusions/Future Plan

Implantable devices have been used to provide the clinical foundations for treating the health conditions of patients suffering from critical neural disorders. Up until now, various recording devices for measuring biological signals, such as electrical activity, optical responses, pressure, temperature, pH, DA, and AA, have been developed to implement advanced diagnosis and feedback system. Biopotential sensors that interrogate signals from the heart, brain, or other organs also assist in monitoring the neuro‐dynamic phenomena by powering systems (implantable battery and energy harvesters). Beyond the conventional recording devices, devices for neuromodulation using small amounts of current, light, or drug delivery leverage neuroscience research with therapeutic devices. However, identifying complex neural circuits is still limited by several challenges posed by designing implants with unique properties.

In this paper, we discussed the problems of implantable devices in six detailed subdivisions: 1) chronic implants guaranteeing long‐term experiment, 2) bioresorbable electronics that eliminate the need for a secondary surgery, 3) an actively multiplexed electrode array to achieve high spatiotemporal biosignal recording, 4) transparent electronics for simultaneous electrophysiological recording and optical modalities, 5) wireless systems for easily conducting behavioral experiments without external noise, and 6) an integrated multifunctional device for successful closed‐loop control of neural systems.

Ultimately, next‐generation medical implants must be developed through a combination of all the concepts and technologies that are presented here. Moreover, a chronic recording/stimulation or transient operation with superior spatiotemporal resolution is required. Only then can advantages such as low‐noise optical modalities, wireless platforms, and multifunctional integration be achieved. The design of next‐generation implantable neural devices with the aforementioned considerations will open up new opportunities not only for clinical applications but also for next‐generation brain machine interfaces or brain computer interfaces by paving the way for communications between prosthetics and the brain.

## Conflict of Interest

The authors declare no conflict of interest.
